# Minimally Invasive Therapies for Female Stress Urinary Incontinence: the Current Status of Bioinjectables/New Devices (Adjustable Continence Therapy, Urethral Submucosal Collagen Denaturation by Radiofrequency)

**DOI:** 10.1100/tsw.2009.53

**Published:** 2009-06-12

**Authors:** Simone Crivellaro, John J. Smith

**Affiliations:** Department of Urology, Wake Forest Medical Center, Winston Salem, NC, USA

**Keywords:** stress urinary incontinence, ACT, collagen denaturation, minimally invasive

## Abstract

The aim of this review is to provide an update on the current status of evolving minimally invasive therapies for stress urinary incontinence. Bioinjectables have been available for some time and their current status is reviewed. The adjustable continence device has been used as a salvage procedure for females for a number of years in clinical trials, yet many are unfamiliar with it. Lastly, radiofrequency via a transurethral route has also been utilized in small numbers and will be updated. These later two emerging technologies need further exposure to better define their role in our clinical practice.

## INTRODUCTION

Stress urinary incontinence (SUI) is a major urologic health problem defined or characterized by the loss of urine during effort, exertion, or other activities such as coughing, sneezing, exercising, or lifting[1]. This condition causes unnecessary and detrimental psychological distress, social isolation, and public expense for care. Surgery remains the cornerstone of treatment for many patients with SUI and is indicated in those who have failed to improve with conservative measures. Many surgical procedures have been described, with varying degrees of success, and the ideal surgical treatment for this condition remains to be determined.

Recent improvements in our understanding of the underlying pathophysiologic mechanisms responsible for SUI in women have led to the development of innovative new surgical methods. Many are less invasive than prior techniques, and appear to offer improved safety and shorter hospital stays, while maintaining the efficacy of traditional open incontinence surgery. Procedures using injectable periurethral bulking agents, insertion of periurethral devices (adjustable continence therapy [ACT]), or urethral submucosal collagen denaturation by radiofrequency (RF) characterize this current trend toward less invasive surgical treatments. The increasing range of available procedures allows surgical treatment of

SUI to be individualized for the patient. Women of diverse ages and levels of medical fitness can increasingly be offered a choice of safe, effective treatment for SUI.

## URETHRAL INJECTABLES

### Introduction

Urethral bulking agents have been used to treat stress incontinence since Murless in 1938, when he used sodium morrhuate or cod liver oil as a sclerosing agent in the anterior vaginal wall of 20 patients, attempting to create an inflammatory response[2]. Others used similar agents, but until 1973, this approach was seldom used. Berg[3] and Politano et al.[4] popularized the use of polytetrafluoroethylene (Teflon®) through the 1970s, however, it was not until the FDA approval of bovine glutaraldehyde cross-linked collagen (Contigen®) that urethral bulking became popular. What follows is a brief review of the materials, their results, and complications.

### Materials

Urethral bulking agents are indicated for urinary incontinence secondary to poor urethral function. This usually means a low Valsalva leak point pressure and limited urethral mobility. Support is a key factor in consideration of urethral bulking. Specifically, if there is poor vaginal support and significant mobility of the bladder neck/urethral complex, other options usually have better results. Of course, a gray area exists, where patient and physician agree to use these agents, fully anticipating a partial improvement in an otherwise poor surgical candidate.

The procedure can be performed through a transurethral[5] or periurethral approach[6]. The former is far more popular. There are two transurethral techniques: the obvious visual approach and a “blind” technique. The nonendoscopic approach was designed ostensibly to eliminate operator variability. First described by Henalla et al.[7], the device was developed to control the placement of the injected material in predetermined areas. This approach was also adopted by the makers of the IMPLACER™ to use dextranomer hyaluronic acid (Zuidex™)[8] (Fig. 1).

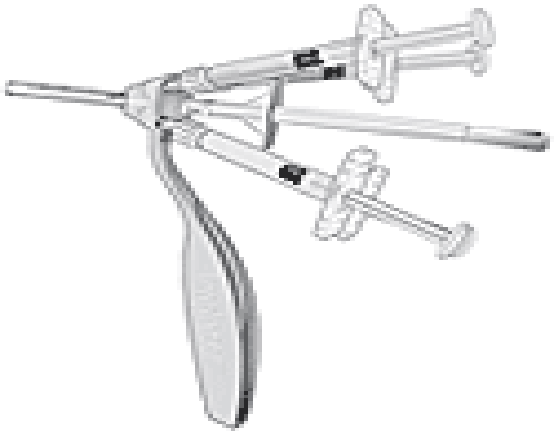

**FIGURE 1.** Implacer device.

Unfortunately, since the FDA trial comparing Zuidex to collagen had a negative result, the company has withdrawn the device from the market[9].

The ideal bulking agent should be biocompatible, easy to use, safe, nonmigratory, noncarcinogenic, have no immunogenic properties, and be permanent, but easily explanted if necessary. A tall order and, indeed, none of the following agents meet those requirements completely.

Polytetrafluorethylene (Teflon, Polytef) is a thick paste material that consists of variable-sized particles (most less than 50 µm, but some 300 µm) with high viscosity, requiring a caulking-type gun to inject the material. The size of the particles permits possible phagocytosis, resulting in migration, which has been reported[10,11]. Additional complications reported include inflammation leading to urethral fibrosis, abscess formation, and urethral diverticulum[10]. Politano's technique, however, did offer men who were postprostatectomy an option in an era prior to the full development of the artificial urinary sphincter. This set the stage for the much-anticipated introduction in 1993 of bovine glutaraldehyde cross-linked collagen (Contigen).

Contigen is a highly purified suspension of bovine collagen in normal saline containing 95% type 1 collagen and 5% type 3 collagen, cross-linked with glutaraldehyde for stability, durability, and reduction in hypersensitivity. A skin test is required prior to injection and a recommended 30 days must pass before treatment. The procedure is simple and easy, and in our hands almost exclusively done in the office. It can be relatively expensive over time, however, and at least one analysis by Berman and Kreder suggested that an uncomplicated sling is more cost effective[12]. Early results showed promise with very high cure and improvement rates[13], but long-term results have been tempered by cure rates of 14–25% and improvement rates of 25–60%[14]. Complications have been divided into early and late. The most common are urgency, transient retention, and hematuria, usually all self-limiting. Other late complications have included delayed skin reactions, particularly with reintroduction of collagen and arthralgia[15]. Serious complications, such as pulmonary emboli and osteitis pubis, have also been reported[16,17]. The material, however, remains the most widely used injectable for SUI in the world.

Durasphere® (carbon-coated zirconium beads) is a sterile, nonpyrogenic, injectable bulking material composed of pyrolytic carbon-coated graphite beads suspended in a 97% water, 3% beta glucan carrier gel. Recently re-engineered via its carrying gel for delivery, it is now available as Durasphere EXP and has an optimized bead size of 90–212 µm. It is safe and has no risk of allergy. Results have been comparable to collagen. Its immediate complications are slightly higher also and are reflected in the rate of urinary retention, which rarely persists or needs surgical intervention[18].

Macroplastique® is a soft, flexible, irregular material made of vulcanized polydimethylsiloxane or simple silicone rubber! The silicone particles are inert, biocompatible, nonbiodegradeable, and nontetratogenic. The mean particle size is 100–300 µm, limiting the chances of migration. The material, however, still must be injected with a special injection system because of its high viscosity. The material was recently approved by the FDA for treatment in SUI.

Results reported as cure and improvements vary from 60 to 80%, respectively[19,20]. Complications are similar to other agents and include transient hematuria, retention, and urinary tract infection (UTI). Barrett and others have expressed concern about small particle migration, but this is certainly not common[21].

Calcium hydroxylapatite, or Coaptite®, is a synthetic injectable implant composed of smooth calcium hydroxylapatite bioceramic microspheres that have a diameter range of 75–125 µm, suspended in an aqueous gel carrier. A normal constituent of bone, it is also nonimmunogenic, previously used in dental and orthopedic surgery. A 12-month prospective, randomized, comparative, multicenter, single-blind, parallel, clinical trial of calcium hydroxylapatite and collagen for soft-tissue augmentation of the urethral sphincter in the treatment of SUI enrolled 296 women and reported essentially comparable results with 63 and 57% improvement, respectively. Approved by the FDA in November of 2005, it is used for the treatment of intrinsic sphincter deficiency (ISD). As yet, there are no serious adverse events reported[22].

More recently, the use of ethylene vinyl alcohol copolymer, or Uryx™/Tegress™, came to a halt because of serious adverse events reported to the FDA. The injectable material previously used to inject arteriovenous malformations was withdrawn from the market in December of 2006, two brief years after its introduction. A large, multicentered trial was reported by Dmochowski et al. with encouraging early results[23], however, the complications of dysuria and pain later developed into erosions and inflammation, often necessitating surgical correction[24].

There have also been attempts to use other materials, including dermal collagen implants, autologous fat, and even autologous chondrocytes[25]. The most exciting and promising field may be that of tissue engineering. There have been some successes using autologous myoblasts and fibroblasts for urinary incontinence[26]. Three-dimensional ultrasounds revealed an increase in the size of the urethra and rhabdosphincter from baseline. Although lately these results have been seriously questioned[27], this could represent another area of future exploration.

In summary, there have been many materials injected in attempts to treat urinary incontinence. Many show early promise, only to disappoint later, and none have long-lasting effect. They do, however, have a role in the urologist's armamentarium. Fortunately, complications are few and usually minor. The best hope is for some breakthrough in the field of tissue engineering, which may allow a simple flexible solution that addresses the need for urethral mucosal support, muscular integrity, and perhaps enhanced vascularity. A tall order!

## ADJUSTABLE CONTINENCE THERAPY (ACT®)

### Introduction

Surgical management of intractable female SUI can be complicated by the increasing presence of scar tissue following each intervention, and the pendulum effect between incontinence and voiding dysfunction. The ACT® (Adjustable Continence Therapy) is a new device for the treatment of female SUI due to ISD. The ACT (Uromedica, Plymouth, MN) was developed in order to address the need for a minimally invasive procedure that could act in a similar way to bulking agents, but without the migration issues of free-floating bulking agents. It was also clear that an adjustable technique was needed for postoperative correction in patients who were not completely continent following surgery and/or in those who developed voiding difficulties as a result of overcorrection. The ACT consists of two silicone balloons attached via conduits to a titanium and silicone port (Fig. 2). The balloons are placed under fluoroscopic image in the periurethral space at the bladder neck, with the aim of increasing urethral resistance and supporting the bladder neck. The ports are buried in the subcutaneous tissue of the labia majora and enable postoperative titration of the balloon should this be required. The procedure is particularly pertinent to those surgeons who are adept with endourological procedures, as an endoscopic dexterity associated with a thorough anatomical understanding is an integral requirement for successfully mastering optimal balloon placement, as well as ensuring improvement in continence. In principle, the ideal female patient is one who has failed primary, secondary, or even tertiary procedures, and has a modicum of scar tissue that may serve to enhance the bulking effect of the balloons. These patients are usually characterized by an open bladder neck or a fixed open proximal urethra at rest, demonstrated on filling phase of a voiding cystourethrogram (VCUG) with a low Valsalva leak point pressure (VLPP) and low maximum urethral closure pressure (MUCP) at urodynamic investigation.

### The Surgical Procedure

With the patient in standard lithotomy position, the bladder is filled with 150 ml of dilute contrast, through an 18Fr Foley catheter, which is inserted with 10 cc of concentrated contrast to enable visualization of the bladder neck. Bilateral, small, 1-cm incisions are made in the labial sulcus at the level of vaginal introitus below the urethral meatus. Using fluoroscopic guidance and digital vaginal palpation, a sharp trocar (Fig. 3) encased in a U-shaped cannula is directed through the incision, perforating the pelvic floor towards the bladder neck. A rotating action employing the blade mechanism on the distal tip

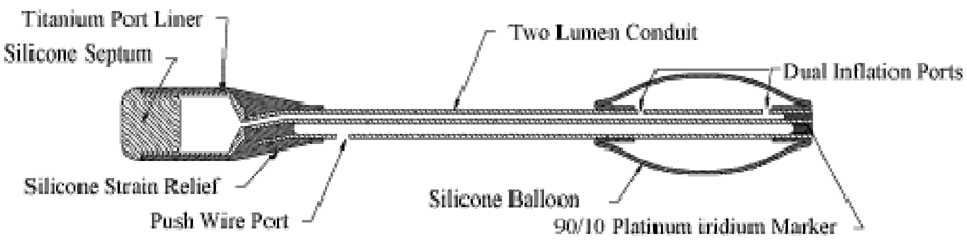

**FIGURE 2.** ACT device.

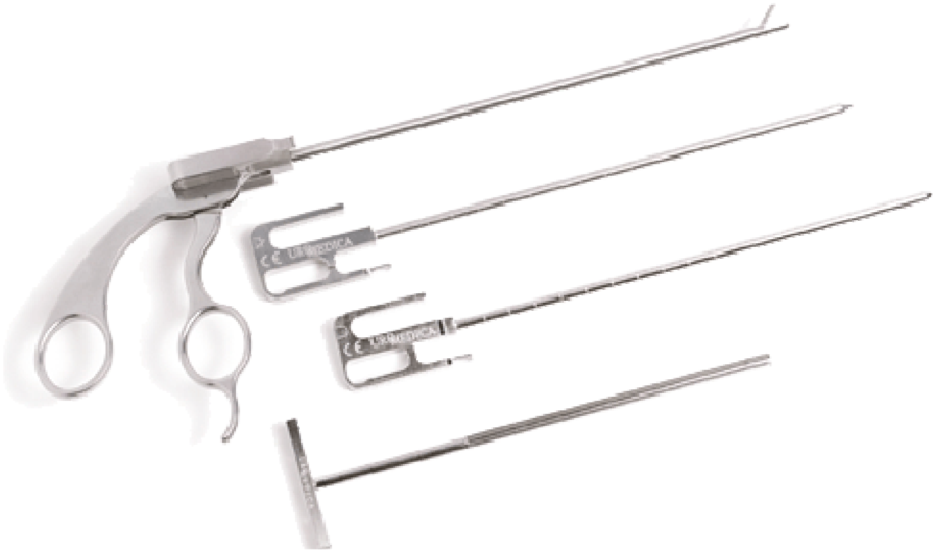

**FIGURE 3.** Inserting trocar for ACT.

of the trocar is used to advance the instrument, while simultaneously using gentle traction on the Foley catheter to locate the bladder neck. Accurate localization of the trocar is assisted by digital palpation against the anterior vaginal wall. Following perforation of the pelvic floor, a blunt trocar may be employed if there is a risk of urethral or bladder perforation. Once the tip of the trocar is at the bladder neck, the trocar is removed and the incremental markers are used to measure the correct device length by retracting the inner trocar, while maintaining the U-shaped channel in position. When the tip of the trocar reaches the labial skin edge, the device length can be determined by counting the corresponding incremental markings at the distal portion of the trocar. The trocar is then fully removed, leaving the cannula in place. The sterile balloon is then primed to remove air and ensure patency. The devices are soaked in antibiotic solution before insertion. The balloon is then lubricated and advanced through the U-shaped channel to the bladder neck, using the removable guide wire as a pusher. Care is taken to avoid undermining the bladder neck and placing the balloon under the bladder trigone as this could result in *de novo* urgency. In the event that a bladder or urethra perforation has occurred, urine will be seen down the U-shaped channel and leakage of contrast solution from the bladder will be observed on fluoroscopy. Following a bladder perforation, the instrumentation should be removed and a new tract created. Placement of the balloon on the side of a urethral perforation should be postponed for 6 weeks to allow for healing and to avoid the possibility of a fistula formation. The balloon is inserted into the U-shaped cannula using a preloaded guide wire as a pusher. The balloon is filled with 1–1.5 cc of an isotonic solution (sterile water and contrast) to stabilize its position and the process is repeated on the contralateral side. The correct position is confirmed on the image-intensification screen and this image should be saved for future reference (Fig. 4). The guide wire and cannula are removed, and the ports are buried in the labia majora in an easily accessible subcutaneous pouch and the incision closed in two layers. The urethral catheter remains *in situ* overnight. Patients are prescribed a prophylactic, single, perioperative dose of gentamycin and a postoperative course of oral ciprofloxacin 500 mg, once daily for 5 days. First adjustments should not be conducted before 4–6 weeks to allow for pseudoencapsulation to mature and subsequent increments should be spaced at a minimum of 4 weeks apart. While incremental adjustments normally are conducted within the first 6 months, titrations can be performed at any time.

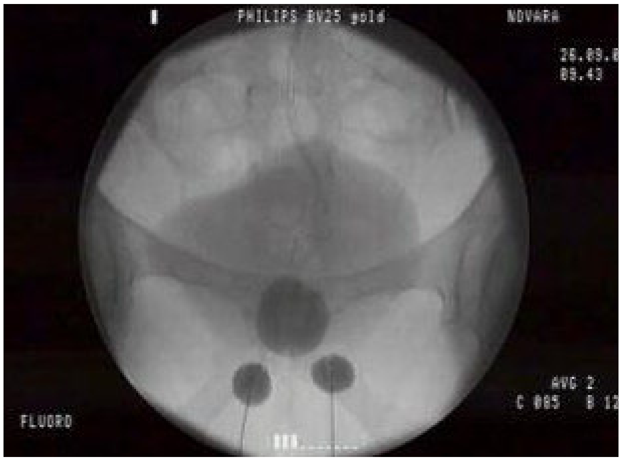

**FIGURE 4.** Cystogram showing a correct location of the ACTs.

## Results

ACT is becoming a well-established treatment option for ISD in Europe. Kocjancic et al.[28] from Udine, Italy, report a series of 49 patients who had previously failed anti-incontinence surgery. Each patient was implanted with the ACT device, and assessed by pre- and postoperative objective and subjective tools. Of the 49 subjects, 38 had a minimum of 1 year data, including pad use, number of adjustments, and complications. Operative time was 20.3 (10–30) min, with 88% of implantations performed using local or regional anesthesia. Balloon adjustments were needed in 62%. All 49 patients were counted in the complications, erosions, and explants. However, 38 patients with at least 12 months of follow-up were evaluated for efficacy. In doing so, 26 patients out of 38 (68%) reported being dry, six out of 38 (16%) improved, and six out of 38 (16%) were unchanged at last follow-up. Postoperatively, there was significant improvement in both pad usage (from 5.3 to 0.1) and quality of life over time (from 31.4 to 93.8). Additionally, patients reported significant improvement in symptoms based on the visual analogue scale (VAS). Complications included migration (12%), balloon failure (3.6%), and erosion (4%).

The French multicenter clinical trial[29] reported 68 patients with SUI who underwent the ACT procedure. The mean operating time was 31.8 ± 11.7 min. The ACT was implanted with no particular difficulties in 91% of cases. Evaluation of patients (mean follow-up: 2 years) after implantation revealed a marked improvement of incontinence (87%). The ACT was removed in 18 patients for various reasons and was reimplanted in six cases. The ACT was removed in eight patients at their request due to complete absence of efficacy.

Finally, the North American ACT trial[30] reported 161 subjects with 1 year of data available on 137 patients. Mean Stamey score improved by >1 in 76.6% of patients (82/107). Significant improvement was shown via the mean Incontinence Quality of Life (I-QOL) Questionnaire (70.5 at 1 year vs. 36.0 at baseline; $p < 0.001$). Additionally, reductions shown in the mean Urogenital Distress Inventory (32.7 at 1 year from 61.3 at baseline; $p < 0.001$) and Incontinence Impact Questionnaire (23.4 at 1 year from 54.2 at baseline; $p < 0.001$) scores were noted. Mean provocative pad weight decreased from 49.7 g at baseline to 11.9 g at 1 year ($p < 0.001$). Device- or procedure-related adverse events were reported in 56.2% of subjects. Of these, 81% were considered to be for mild severity. The mean number of balloon volume adjustments through 1 year was 2.1 (0–8).

We summarized the results of the three studies in Table 1.

**TABLE 1**
**Reported Results and Complications of ACT**

| | No. of Patients | Follow-Up (Months) | Dry + Improved (%) | Removal (%) |
|---|---|---|---|---|
| Kocjancic et al. | 49 | 28 | 84 | 20 |
| French group | 68 | 24 | 76 | 26 |
| American group | 137 | 16 | 77 | 56 |

## Discussion

The concept of using injectables to increase urethral resistance is well established. Two of the known complications of injectables are the difficulty in ensuring accurate placement of the material and the prolonged maintenance of effect[31,32,33,34]. Possible operative complications relating to the ACT include bladder or urethral perforation. Bladder perforations are easily managed by the creation of a new tract and extended postoperative catheterization of 3–5 days. In the case of a urethral perforation, balloon implantation on the affected side should be postponed for 6–8 weeks. Potential postoperative complications are primarily balloon migration, urethral erosion, and infection. Migration may be related to a poor surgical technique or to previous anti-incontinence surgery. Some implanters report that positioning of the ACT device is more challenging in the presence of a midurethral, tension-free tape placed either with a retropubic or transobturator approach than it is following injection of bulking agents or open suspension procedures, and they suggest that in these cases, the ACT device should be placed cranially to the tape. According to Kocjancic et al., erosion secondary to infection is a complication that can be limited by the judicious use of antibiotics perioperatively, and they stress the importance of utilizing aseptic techniques during implantation and subsequent adjustments. To date, no late infections have occurred as can be seen with other urologic prostheses. Interestingly, postoperative complications are minor and can be easily treated by device removal, which can be performed in an outpatient setting.

Unlike many other surgical techniques that demonstrate a high short-term success that decreases over time[7], results of the ACT appear to improve over time. This may be a result of the ability to adjust the pressure balloon even at a very long follow-up (up to 3 years), as Kocjancic et al. reported.

In conclusion, the ACT device and technique provide a good outcome in a difficult group of patients, and seems an appropriate second- or third-line therapy best suited for women seeking continence after having failed other procedures.

## TRANSURETHRAL SUBMUCOSAL COLLAGEN DENATURATION (RENESSA®)

## Introduction

A treatment option recently approved by the FDA to treat patients who have SUI due to hypermobility can be administered in approximately 30 min in an outpatient or office setting using local anesthesia[35].

This nonsurgical, transurethral RF energy (RFe) collagen denaturation system (Renessa, Novasys Medical, Inc., Newark, CA) administers RFe through a transurethral probe to induce submucosal collagen denaturation and reduction in regional tissue compliance. This treatment greatly differs from an older RF treatment, the surgical transvaginal RF ablation procedure (SURx Transvaginal System, Cooper Surgical, Trumbull, CT). The transurethral RF denaturation procedure does not produce tissue necrosis, while the RF ablation procedure intentionally destroyed cells to produce gross shrinkage or vascular destruction[36,37,38,39]. Rather, RF collagen denaturation aims to reduce regional dynamic tissue compliance without creating strictures or reducing luminal caliber, resulting in a functional rather than anatomic change[40,41,42].

## Technique

The procedure is usually carried out on an outpatient basis in the clinical procedure room, cystoscopy suite, or outpatient operating room at each study site. The patient is placed in the lithotomy position. Sedation and analgesia are usually achieved through the intravenous administration of midazolam and fentanyl, with or without propofol. Antimicrobial prophylaxis is usually performed by ciprofloxacin 500 mg intravenous within an hour after the procedure begins. Lidocaine gel is applied topically to the RF probe prior to insertion. A standard grounding pad is placed on the patient's body and the device is connected to the RF generator using a power cable. A bag of sterile water (1 l) kept at room temperature is connected to the generator by means of intravenous tubing. A foot pedal is also connected to the generator. A standard urinary catheter is inserted through the urethra into the bladder and the bladder is emptied. Room-temperature water (30 cc) is instilled into the bladder through the catheter, which is then removed. The RF device is then inserted through the urethra into the bladder. The balloon in its distal tip is inflated within the bladder lumen using 10-cc sterile room-temperature water and positioned within the bladder outlet by palpation (Fig. 5).

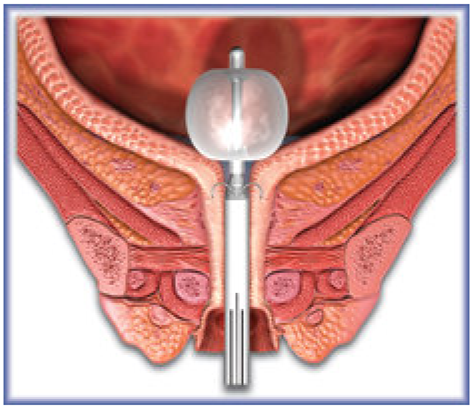

**FIGURE 5.** Radiofrequency device.

The generator is set to deliver RFe in nine cycles of 60 sec each. During each 60-sec cycle, the device creates a set of four lesions in the bladder neck and proximal urethra submucosa. After each set of lesions has been made, the device is repositioned. The temperature of mucosal and submucosal tissue and the tissue impedance is monitored throughout the procedure. Whenever any of these readings exceeds established safety levels, the device needles automatically stop delivering RFe; they are then withdrawn, the balloon deflated, and the device removed.

## Results

Collagen denaturation treatment for SUI is a relatively new concept. Appell et al.[43] reported results on a total of 21 patients who underwent RF and were evaluated after 3 years with completed 3-day diaries. Diaries were reviewed to assess the number of patients with at least a 50% reduction in IEF (incontinence episode frequency) compared with baseline. The change from baseline in the overall I-QOL score was calculated for the 21 participants who had not undergone additional SUI intervention. The mean baseline I-QOL score was 56.3 ± 24.6. Evaluation more than 3 years after the procedure revealed a mean improvement of 12.7 ± 26.0 points (range: –26.14 to 62.5; $p = 0.04$).

Overall, 18 patients had 3-day diaries available for analysis at more than 3 years post-treatment. After 3 years, 50% of all patients included in the IEF analysis had achieved a 50% or greater reduction in IEF. It should be noted that although patients with leaks associated with urge urinary incontinence (UUI) were excluded from the original trial, some study patients reported intermittent urge leaks, but no patients developed UUI requiring treatment. When excluding leaks marked in diaries as associated with UUI, 56% of patients reported at least a 50% IEF reduction. No serious adverse events occurred during the initial 12-month trial period, and there were no significant differences in adverse events between the treatment and sham arms. No new adverse events were reported after 3 years.

Sotomayor and Bernal[44] reported 41 women treated by RF for SUI divided into four different subgroups. They were codified by the number and lower urinary tract location of microremodeling sites. The four treatment groups did not differ statistically in terms of patient age, duration of incontinence, or IEF. Thirty-six women were available for analysis at 12 months following RF microremodeling.

The incidence of I-QOL overall score improvement and the mean score improvement for each treatment group at 12 months are presented in Table 2. The incidence of >10-point improvement in I-QOL overall score for each treatment group at 12 months is also presented in Table 2. The incidence of improvement and mean score improvement for the three I-QOL subscale scores for each treatment group at 12 months is presented in Table 3. IEF data were analyzed to determine the incidence of any IEF reduction, the incidence of >50% IEF reduction, and cure at 12 months following treatment (''cure'' is defined as no incontinence episodes and no use of incontinence pads between 6 and 12 months). Results of these IEF reduction analyses are presented in Table 4. IEF reduction was statistically significant ($p < 0.05$) at 12 months vs. baseline for treatment groups I, II, and IV.

**TABLE 2**
**Improvement at 12 Months in I-QOL Overall Score vs. Baseline**

| Treatment Group (*n*) | I-QOL Score Improvement Incidence (%) | Mean I-QOL Score Improvement ± SD (*p* Value) | >10-point I-QOL Score Improvement Incidence (%) |
|---|---|---|---|
| I (8) | 75 | 23 ± 29 (NS) | 63 |
| II (9) | 78 | 16 ± 21 (0.05) | 44 |
| III (10) | 70 | 17 ± 22 (0.04) | 70 |
| IV (9) | 78 | 24 ± 27 (0.03) | 67 |

Through 12 months, there remains a 0% incidence of serious adverse events. Between the 6- and 12-month follow-up visits, 8% of patients experienced at least one episode of dysuria and 22% admitted to at least one episode of urgency, both anticipated adverse events. None of these adverse events was deemed by a patient as being problematic in terms of quality of life.

**TABLE 3**
**Improvement at 12 Months in Three I-QOL Subscale Scores vs. Baseline**

| Treatment Group (*n*) | Incidence of I-QOL Subscale Score Improvement (%) | Improvement in Mean I-QOL Subscale Score ± SD (*p* Value) |
|---|---|---|
| Avoidance and limiting behavior I-QOL subscale | | |
| I (8) | 75 | 18 ± 34 (NS) |
| II (9) | 78 | 18 ± 29 (NS) |
| III (10) | 80 | 23 ± 25 (0.01) |
| IV (9) | 67 | 23 ± 33 (NS) |
| Psychosocial impact I-QOL subscale | | |
| I (8) | 75 | 23 ± 26 (0.04) |
| II (9) | 56 | 9 ± 17 (NS) |
| III (10) | 70 | 8 ± 22 (NS) |
| IV (9) | 67 | 20 ± 29 (NS) |
| Social embarrassment I-QOL subscale | | |
| I (8) | 88 | 32 ± 36 (0.04) |
| II (9) | 56 | 24 ± 27 (0.03) |
| III (10) | 80 | 24 ± 24 (0.01) |
| IV (9) | 78 | 32 ± 29 (0.01) |

**TABLE 4**
**IEF Reduction and Cure at 12 Months**

| Treatment Group (*n*) | Incidence of IEF Reduction (%) | Incidence of >50% IEF Reduction (%) | Incidence of Cure (%) |
|---|---|---|---|
| I (8) | 88 | 63 | 40 |
| II (9) | 67 | 67 | 22 |
| III (10) | 70 | 70 | 40 |
| IV (9) | 89 | 89 | 67 |

## Discussion

Transurethral delivery of RFe is an appealing nonsurgical treatment option for SUI in that it is performed in the outpatient setting under conscious sedation, and without the need for cystoscopy or fluoroscopy. Furthermore, the technique is rapidly learned and easily performed. The initial clinical trials performed demonstrated device and procedural safety, as well as improvement in patient quality of life at 3 years following treatment.

No serious adverse events have been reported thus far. Elicited “urgency” events are mainly sensory in nature, without associated urge incontinence, and few required anticholinergic therapy.

In addition, given the miniscule volume of tissue actually remodeled within the vesicourethral junction and proximal urethra, it is unlikely that RFe microremodeling will result in the development of a “lead pipe” urethra. Reports more than 1 year after RFe remodeling of the esophagus and at 1 year following RFe remodeling of the anal canal have demonstrated neither “lead pipe” changes nor other chronic safety concerns[45]. However, again because of the limited volume of tissue microremodeled, repeat treatment is likely and while probably a safe, viable option, it will need to be studied further.

Additionally, the limited nature of RFe microremodeling probably does not inhibit future alternative surgical or nonsurgical incontinence therapy.

Although no randomized control trials are available, RFe microremodeling seems an appropriate first-line therapy best suited for women seeking an improved quality of life in exchange for nonsurgical therapy, minimal risk, rapid recovery, the potential for repeat treatment, and the capability to undergo alternative future therapies.

## CONCLUSION

Minimally invasive therapies for SUI are available for us to utilize, yet their long-term efficacy is limited. Injectables remain the main choice of most urologists for minor incontinence or patients not amenable to surgery. The results of the ACT device look very promising. RFe has not been used in great numbers and longer results are awaited. Lastly, tissue engineering may offer the greatest potential for future success.
